# Expression of the immune checkpoint CD137/CD137L in oral squamous cell carcinoma (OSCC) and association with histopathological parameters

**DOI:** 10.1007/s00428-026-04430-4

**Published:** 2026-02-24

**Authors:** Jacek Glajzer, Jutta Ries, Leah Trumet, Christian Matek, Marco Kesting, Manuel Weber

**Affiliations:** 1 Department of Oral and Cranio-Maxillofacial Surgery, Friedrich-Alexander-Universität Erlangen-Nürnberg (FAU), Glueckstrasse 11, 91054 Erlangen, Germany; 2 Institute of Pathology, Friedrich-Alexander-Universität Erlangen-Nürnberg (FAU), Erlangen, Germany; 3 Department of Operative Dentistry and Peridontology, Friedrich-Alexander-Universität Erlangen-Nürnberg (FAU), Erlangen, Germany

**Keywords:** Oral squamous cell carcinoma, Tumor microenvironment, Tumor immunology, Immunohistochemistry, RT-qPCR, CD137

## Abstract

As immunotherapy (IT) with checkpoint inhibitors continues to make rapid progress in the treatment of oral squamous cell carcinoma (OSCC), response rates still necessitate substantial improvement. Current concepts to improve therapy response mainly consider identification of further immunological targets eligible for extension of IT. One auspicious immune checkpoint is CD137 and its ligand CD137L, but expression rates and relevance for progression of OSCC are still unknown. Tissue and peripheral blood from 159 OSCC patients and 55 healthy oral mucosa (HOM) controls was collected and subsequently analyzed for expression of CD137 and CD137L by quantitative reverse transcription polymerase chain reaction (RT-qPCR). Furthermore, OSCC and HOM tissue samples from both groups were evaluated for CD137 protein expression by immunohistochemistry (IHC). Expression rates were analyzed and subsequently correlated with histomorphological characteristics. CD137 exhibited highly significant upregulation in OSCC tissue on mRNA and protein level, as evidenced by both RT-qPCR (p < 0.001) and IHC (p < 0.001) analysis. Regarding the CD137L expression in the examined tissue specimens, there was no significant difference detectable on mRNA level (*p* = 0.53). Comparative analysis of RT-qPCR blood data showed no statistically significance in expression rates of CD137 (*p* = 0.653) and CD137L (*p* = 0.351). Correlation analysis revealed no highly significant results with reference to the histopathological parameters. CD137 showed a highly significant upregulation in OSCC tissue compared to the HOM control group. Consequently, it may be a relevant checkpoint involved into formation and progression of OSCC. Further investigations are needed to evaluate, if CD137 represents a suitable target for a novel IT approach in OSCC treatment.

**Supplementary Information:**

The online version contains supplementary material available at https://doi.org/10.1007/s00428-026-04430-4.

## Introduction

At present, head and neck squamous cell carcinoma (HNSCC) represents one of the most prevalent and lethal human malignancies on a global scale. In the year 2022, GLOBOCAN documented 389,846 new cases of HNSCC affecting the lip and oral cavity, in addition to 188,438 deaths worldwide [1]. According to the current German treatment guideline, first line therapy for patients with newly diagnosed, resectable Oral Squamous Cell Carcinoma (OSCC) should consist of surgical resection with lymphatic node management, potentially augmented by adjuvant (chemo-)radiation [2]. Alternatively, primary (chemo-)radiation may be applied in compromised patients or unresectable tumors [2]. Despite fundamental progress of surgical techniques over the last decades, especially due to introduction of microsurgical free flap transfer, patients’ prognosis did not improve relevantly [3]. It is therefore evident that further therapeutic options and tools are needed to enable the individualization of the optimal treatment regimen for each patient [4]. In this context, immunotherapy (IT) with checkpoint inhibitors, especially Programmed Cell Death Protein-1 (PD-1) inhibitors, expanded the range of therapeutic options. In light of the recently published KEYNOTE-689 study, which met its primary endpoint of event-free survival in newly diagnosed, locally advanced HNSCC patients treated with neoadjuvant and adjuvant pembrolizumab, further and earlier indications for PD-1 inhibitors are anticipated in the future [5]. However, optimal efficiency of PD-1 inhibitors is mostly limited to patients with high expression of PD-L1. Therefore, response rates currently fluctuate between only 14.6–30.1% [6, 7]. As oral squamous cell carcinomas (OSCC) are generally characterized by a highly immunosuppressive milieu with a large inter-individual heterogeneity of molecular expressions, the identification of further immune checkpoints eligible for combination therapy represents a critical avenue to expand the scope of IT applications [8, 9]. In this context, CD137 and its ligand CD137L have become increasingly relevant to cancer research [10]. Differential expression of these markers on mRNA level was furthermore confirmed in a broad NanoString nCounter® assay in OSCC tissue previously performed by our study group [11].

CD137, also known as 4-1BB or Tumor Necrosis Factor Receptor Superfamily member 9, is an inducible receptor mainly expressed on the cell membrane of activated T cells but also a number of other immune cells, including Natural Killer cells (NKs), Dendritic cells (DCs), regulatory T cells (Tregs), Mast cells, Monocytes and other leucocytes [12]. Its ligand, CD137L, is expressed on the surface of antigen-presenting cells (APCs) [12]. After binding of three monomeric CD137s to the trimeric CD137L, an intracellular signaling pathway is induced, resulting in increased activation, survival and differentiation of CD8^+^ T cells [13, 14]. Additionally, CD137 activation was proven to initiate an anti-tumor T cell memory response [15]. CD137 expression has not only been confirmed on immune cells, yet also in various human neural cells and endothelial cells in the tumor microenvironment (TME) [16, 17].

To date, two CD137 agonists, namely Utomilumab and Urelumab, have been established and tested in preclinical and first clinical phase I trials among various hematopoietic and solid tumors with diverging results. While preclinical studies showed very promising antitumor activity and induction of substantial immunological memory for Urelumab, further investigations revealed severe, dose-related hepatotoxicity when applied in humans [18, 19]. It therefore seems evident, that the CD137/CD137L signaling complex represents a promising target for anti-cancer IT, but further adjustment of the optimal agent and dose still need to be determined for possible implementation into therapeutic regimens in the future.

Previous studies performed on humans with solid tumors partially included a very small number of HNSCC patients. However, the impact of the CD137/CD137L signaling pathway on OSCC development and progression is still insufficiently understood and needs further clarification. Therefore, it was the aim of the present cross-sectional study to quantify and compare expression levels of the immune checkpoints CD137 and CD137L on mRNA and protein level in tumor tissue and peripheral blood of OSCC patients compared to healthy controls. Additionally, possible association of CD137/CD137L expression levels with clinical presentation and histologic characteristics was examined.

## Material and methods

### Study collective

For this retrospective analysis, tissue and peripheral blood samples were collected from patients with an initial diagnosis of oral squamous cell carcinoma (OSCC subgroup) and patients with healthy oral mucosa (regarding infection and malignancy, HOM subgroup). All patients were treated at the Department for Oral and Cranio-Maxillofacial Surgery and the University Hospital Erlangen, FAU Erlangen-Nuremberg, between 2010 and 2021. Informed consent was obtained from each patient prior to their inclusion in the study, and local ethics committee approval was obtained before the study’s initiation (application number 3962).

The OSCC subgroup tissue was classified by tumor size (T1-T4) and further clustered into groups of smaller-sized tumors (T1 and T2) as well as larger ones (T3 and T4) according to the 2017 TNM classification. Furthermore, lymph node status (N0 vs. N +), invasion of lymphatic vessels (L0 vs. L1), perineural invasion (Pn0 vs. Pn1), and tumor grading (G1-G3) were determined for each patient.

### Processing of tissue and blood samples

All tissue and blood samples from the OSCC and HOM subcohort were collected during primary surgical treatment, as previously delineated by our research group [20]. Prior to IHC staining, formalin-fixed and paraffin-embedded tissue samples were cut into 2-µm-thick sections and subsequently histopathologically examined for TNM classification and sufficient tumor cell count (OSCC patients, > 70% tumor cells) as well as absence of inflammation and dysplasia (HOM patients). The remaining tissue biopsy was stored in RNAlater® solution (Qiagen, Hilden, Germany) and further used for RT-qPCR analysis. Therefore, samples were initially incubated at 4 °C for 24 h, followed by storage at −80 °C until mRNA isolation.

Blood samples consisted of 2,5 ml peripheral blood stored in a PAXgene Blood RNA Tube (PreAnalatytiX GmbH, Hombrechtikon, Switzerland). These samples were then subjected to two hours of incubation at room temperature, followed by a 24-h storage period at −20 °C. Thereafter, the samples were transferred to −80 °C storage until RNA isolation was conducted.

### Isolation of mRNA and analysis of CD137 and CD137L expression by quantitative reverse transcription polymerase chain reaction (RT-qPCR)

Initial tissue disruption was performed using a Precellys® homogenizer (Bertin Instruments Company, Bertin, France). Subsequently, mRNA was isolated using the Qiagen miRNeasy mini-Kit (Qiagen Company, Hilden, Germany) according to the manufacturer’s instructions. The total mRNA quality and quantity was assessed using the Nano-Drop 3.3 ND1000 spectrometer and the associated ND-1000 software (Thermo Fisher Scientific Company in Waltham, MA, USA, v3.8). Reverse transcription of RNA into cDNA was performed using the Transcriptor High-Fidelity cDNA Synthesis Kit according to the manufacturer’s recommendations (Roche, Mannheim, Germany).

To perform a semi-quantitative analysis of CD137 and CD137L expression, gene-specific primers (specifications, see supplementary Table 1) were applied in conjunction with the QuantiTect SYBR® Green PCR Kit (Qiagen, Hilden, Germany) as previously described [21]. The annealing temperature for both primers was set to 60 °C and the number of PCR cycles was limited to 40. The RT-qPCR was performed and analyzed using the Applied Biosystems QuantStudio 6 Pro Real-Time PCR System (Thermo Fisher Scientific Inc., Waltham, MA, USA).

The data were normalized using the ΔCT with GAPDH as an internal control. To analyze differences in expression between the two genes, the ΔΔCT method was applied (RQ = Fold Change [FC] = 2^−ΔΔCT^).

### Detection and quantitative analysis of CD137 expression by immunohistochemistry (IHC)

**Fig. 1:**
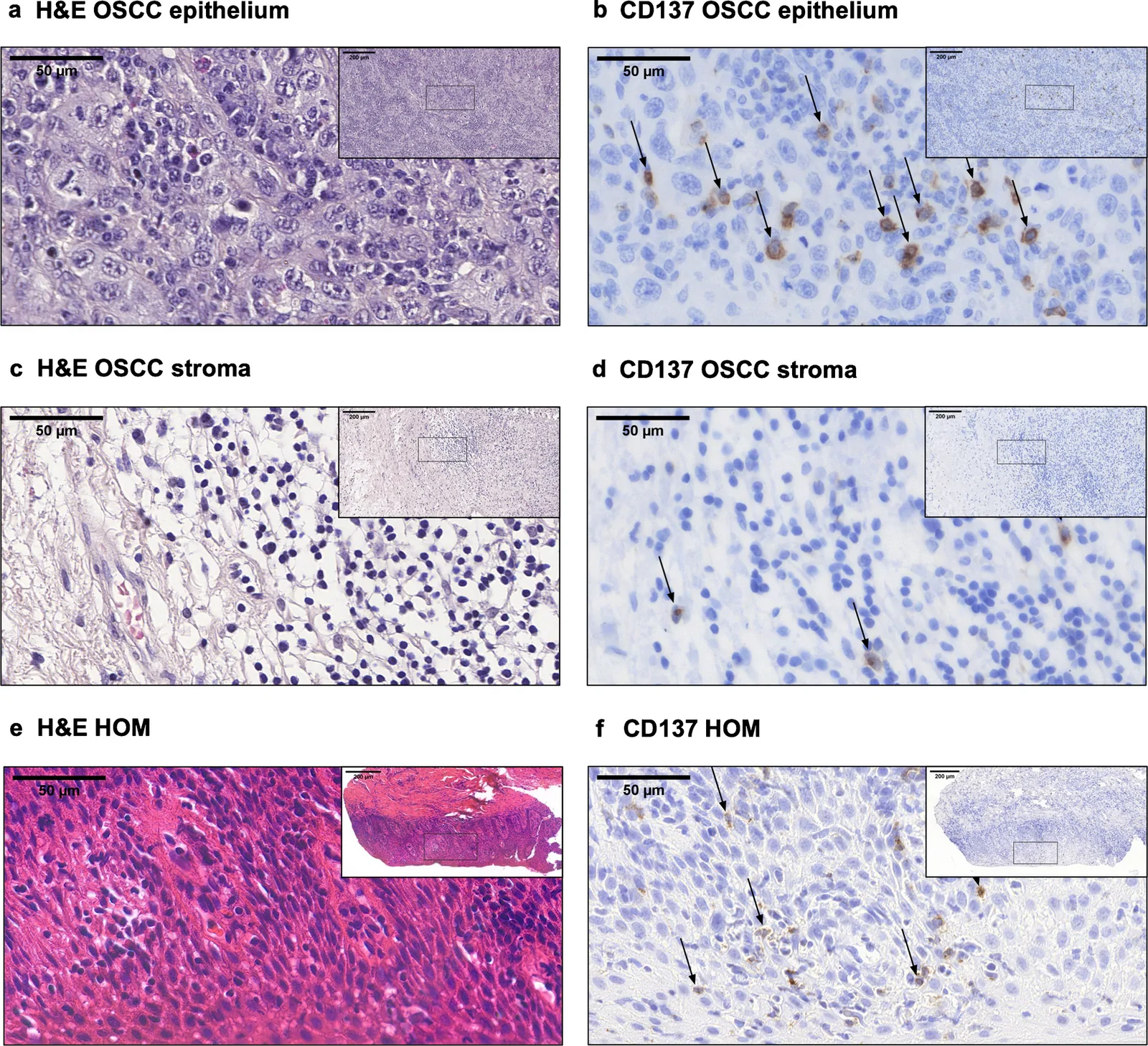
CD137 expression (**b,d,f**) with corresponding hematoxylin and eosin stains (H&E; **a,c,e**) in OSCC tissue and healthy oral mucosa (HOM) as a control group. Exemplary micrographs in high power (50x) and panoramic (10x) magnification showing the typical expression patterns (examples marked by an arrow) of CD137 in epithelial (b) and stromal OSCC compartments (d) as well as HOM (f)

The slices were pretreated with an EDTA buffer at 100 °C in a water bath for 30 min to unmask the epitope. Thereafter, samples were cooled down before being stored in a wash buffer (pH 7,6, DAKO, S3006) for five minutes. The slices were then treated with an anti-CD137 antibody (Abcam, ab252559 rabbit monoclonal, dilution: 1:250) in adherence to the manufacturer’s instructions. Human tonsillar tissue served as a positive control and oral mucosa treated with antibody diluent alone was used as a negative control for reference. Due to poor performance of the CD137L antibody in the testing phase of the study, further staining of all tumor tissue samples was discontinued. Subsequently, slides were digitized using a Panoramic 250 Flash III Scanner (3DHISTECH®, Budapest, Hungary) at 40 × magnification using the whole slide imaging mode at the Department of Pathology, University Hospital Erlangen. Digital cell count was entirely performed using the QuPath open source software for bioimage analysis (version 0.5) [22]. Identical to a previously published examination of our study group, three designated fixed-size (0.5 mm^2^) regions of interest (ROIs) were defined for each tissue sample [20]. The ROIs were subsequently subdivided into the epithelium and the stroma areas. Using the deep-learning based StarDist extension, cell count for each division was executed [23]. Positive cell count was performed manually by two trained scientists and confirmed by a pathologist (Fig. 1). The number of positive cells was then divided by the total cell count of each ROI and multiplied by a factor of 100 in order to determine the labeling index (LI). The average LI of each of the three ROIs was established and used for statistical analysis.

### Statistical analysis

Statistical analysis for the study was conducted using the SPSS 23 software (SPSS Inc., Chicago, IL, USA). To evaluate the RT-qPCR expression between both groups, the ΔΔCT method was employed to calculate the FC. A FC value greater than 2 was considered a relevant increase. To assess CD137 expression based on IHC staining, the mean LI from both groups (LI_OSCC_/LI_HOM_) was utilized as the corresponding factor.

Initially, the data were subjected to a Shapiro–Wilk test to ascertain their distribution. Given the data were not normally distributed, non-parametric statistical tests, specifically the Mann–Whitney U test and Kruskal–Wallis test, were applied to assess the disparities in gene expression. A *p*-value ≤ 0.05 was considered statistically significant.

Receiver Operator Characteristic (ROC) curves were created in order to determine cut-off points (COP) by plotting sensitivity (true positive rate) against specificity (1-specifity, false positive rate). The thereby created area under the curve (AUC) demonstrates the strength of the marker for accuracy of discrimination between two groups, in this case OSCC group and the HOM cohort. Using the ROC-curve, Youden Indices (J = sensitivity – [1-specifity]) were estimated, with the highest index denoting the COP. The COP therefore signifies the point on the given ROC curve that maximizes both sensitivity and specificity. With relation to the COP, the cohorts were again subdivided into patients showing an underexpression of the marker (ΔCT higher than the COP, LI lower than the COP) and patients showing an overexpression of the marker (ΔCT lower than the COP, LI higher than the COP). In a further analysis, correlation of over- and underexpression of the immune checkpoints with other clinicopathological parameters such as diagnosis, TNM-classification and grading was assessed using the Chi-square test (χ^2^-test).

## Results

### Demographic and clinico-pathological characteristics of the study cohort

A total of 159 OSCC patients were enrolled in the RT-qPCR group, while 133 patients were part of the IHC subcohort. Due to inadequate quantity of tissue obtained, not all patients examined in the RT-qPCR group could be evaluated via IHC. In the HOM cohort, 55 patients were analyzed by RT-qPCR and 48 patients by IHC. Mean age in the RT-qPCR subcohort was 62.2 years in the OSCC group and 52.5 years in the HOM group, yielding a statistically significant result (*p* < 0.001). A statistically significant age distribution disparity was observed in both IHC groups as well, with 63.4 years in the OSCC subcohort and 50.3 years in the HOM control group (*p* < 0.001). The demographic and histopathological characteristics of the RT-qPCR and the IHC subcohort are presented in Table 1.

**Table 1:**
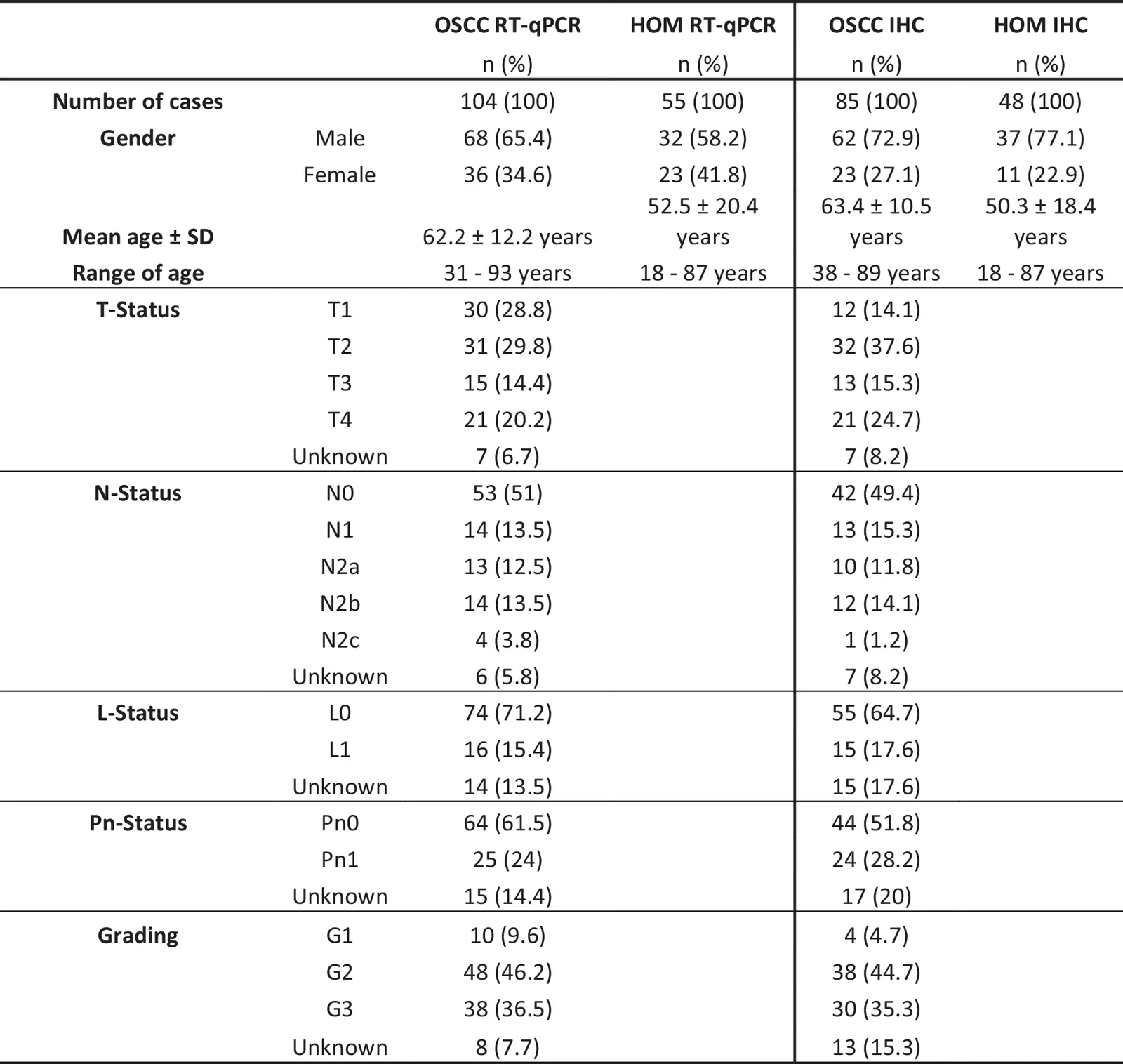
Demographic characteristics of the included OSCC patients and the healthy mucosa controls (HOM), devided by RT-qPCR and immunohistochemistry (IHC). Distribution of T-Status, N-Status, L-Status, Pn-Status and Grading are given for the OSCC subcohort, whereas gender and age characteristics are summarized for both groups

### IHC staining pattern of the CD137 marker

CD137 antibodies showed staining of the cytoplasm and especially the cell membrane as reported before [24]. Majority of positively stained cells had immune cell-like morphology but tumor cells were proven to express CD137 as well (Fig. 1b, d). OSCC tissue samples demonstrated higher overall CD137 expression levels compared to HOM patients (*p* < 0.001). Positive CD137 staining related to the total cell count per compartment, represented by the LI, was higher in the epithelial compartment compared to stromal cells (*p* = 0.002).

### Quantitative comparison of CD137 and CD137L expression in tissue samples of OSCC and HOM patients

#### Immunohistochemistry

**Fig. 2:**
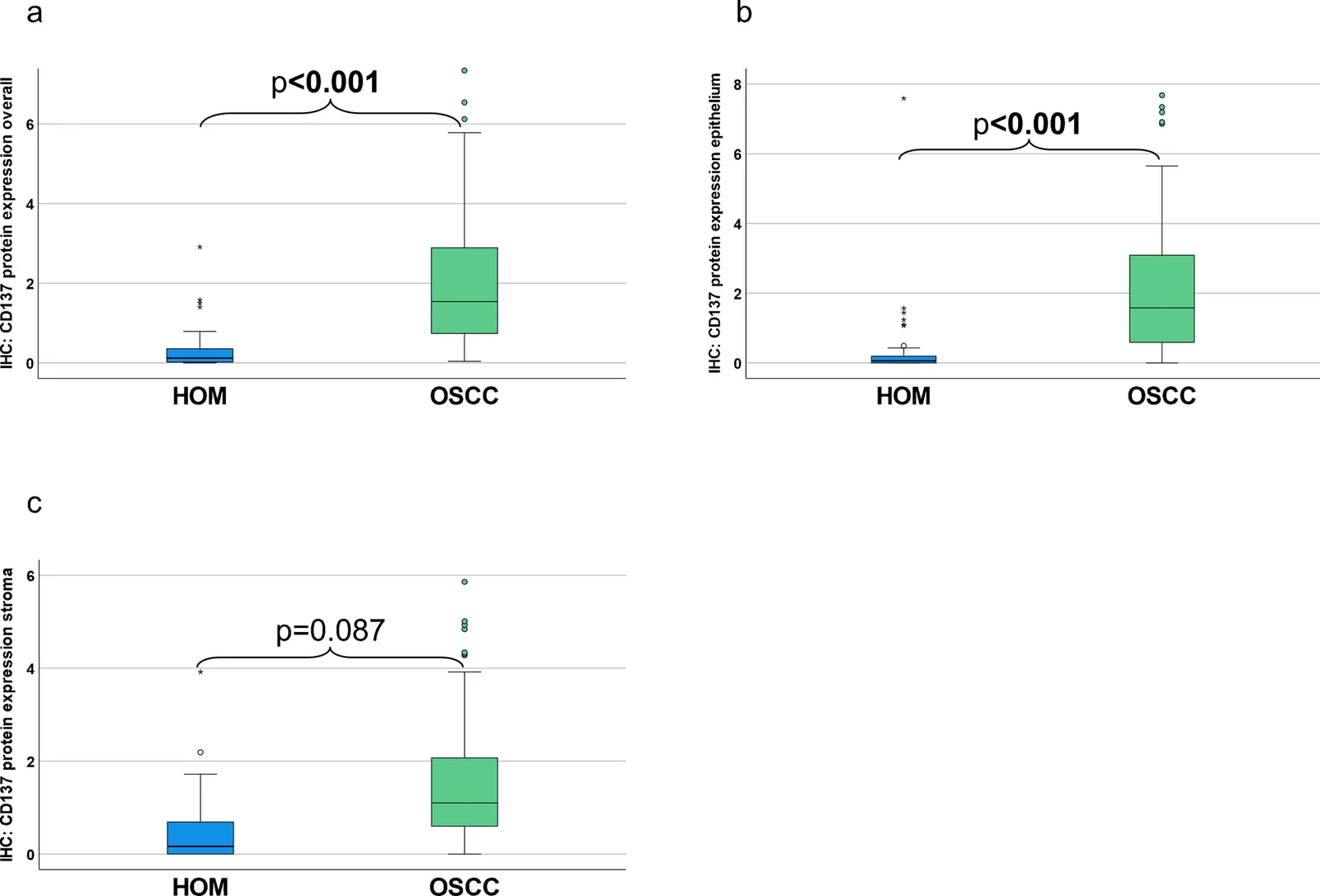
Box plots presenting median protein expression of CD137 in OSCC and HOM by immunohistochemistry. Median labeling indices (positive cells vs. all cells) are presented for the whole analyzed ROI (CD137 expression overall, **a**) as well as per individual cell subclass of cells (CD137 expression epithelium, **b** and CD137 expression stroma, **c**)

CD137 expression levels in tumor tissue were significantly higher (*p* < 0.001) in comparison to HOM tissue, with an average of 61.75 positively stained cells per ROI in the OSCC group (LI = 2.12) vs. 7.32 positively stained cells per ROI in the HOM subgroup (LI = 0.46). Furthermore, a subdivided analysis for the epithelial and stromal compartment was performed. Both compartments showed higher overall CD137 expression rates in the OSCC subgroup (Fig. 2). However, a statistically significant difference was only observed in the epithelial compartment, which contained a mean of 44.25 positive cells per ROI (LI = 2.39, *p* < 0.001) in the OSCC group versus 3.97 positive cells per ROI in the HOM group (LI = 0.37). On the other hand, a mean of 17.4 versus 3.41 positive cells per ROI were counted in the stromal compartment (LI = 0.63 versus 0.97, *p* = 0.087).

#### Quantitative reverse transcription polymerase chain reaction

**Fig. 3:**
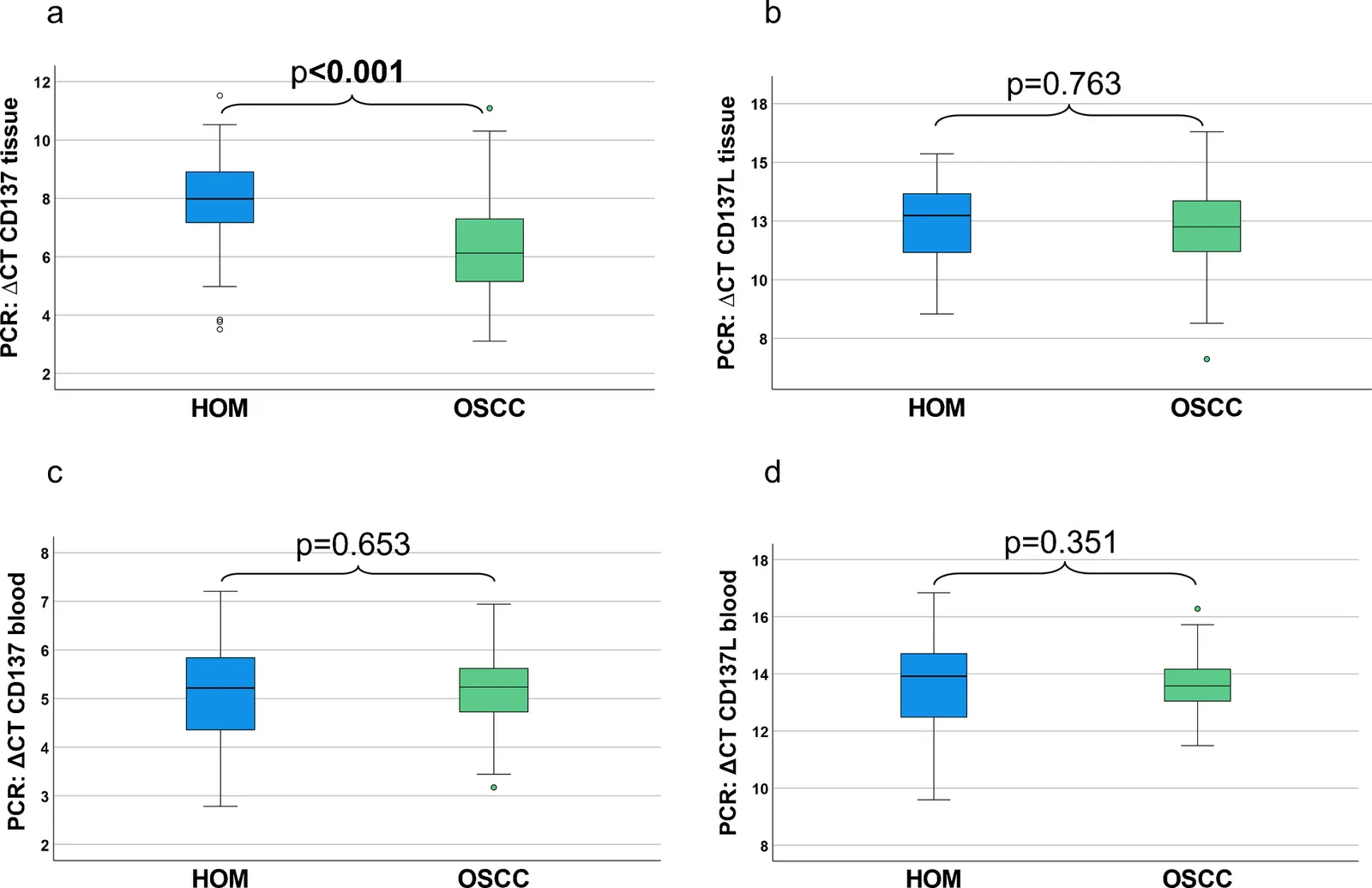
Box plots representing the mRNA expression of CD137 (**a**) and CD137L (**b**) in OSCC and HOM estimated by real-time polymerase chain reaction (RT-qPCR). Low ΔCT levels represent upregulation of mRNA expression, since fewer PCR cycles were needed to detect it. The difference of mRNA expression values is represented by the p-value, statistically significant values are marked by bold numbers

In the RT-qPCR analysis, CD137 demonstrated significantly higher mRNA expression rates in OSCC compared to HOM with a mean ΔCT of 6.32 versus 7.91 (FC = 2.99, *p* < 0.001, Fig. 3a). Conversely, CD137L exhibited no substantial disparities in the expression rates, with a mean ΔCT of 12.28 in OSCC vs. 12.38 in the HOM cohort (FC = 1.06, *p* = 0.763, Fig. 3b).

**Fig. 4:**
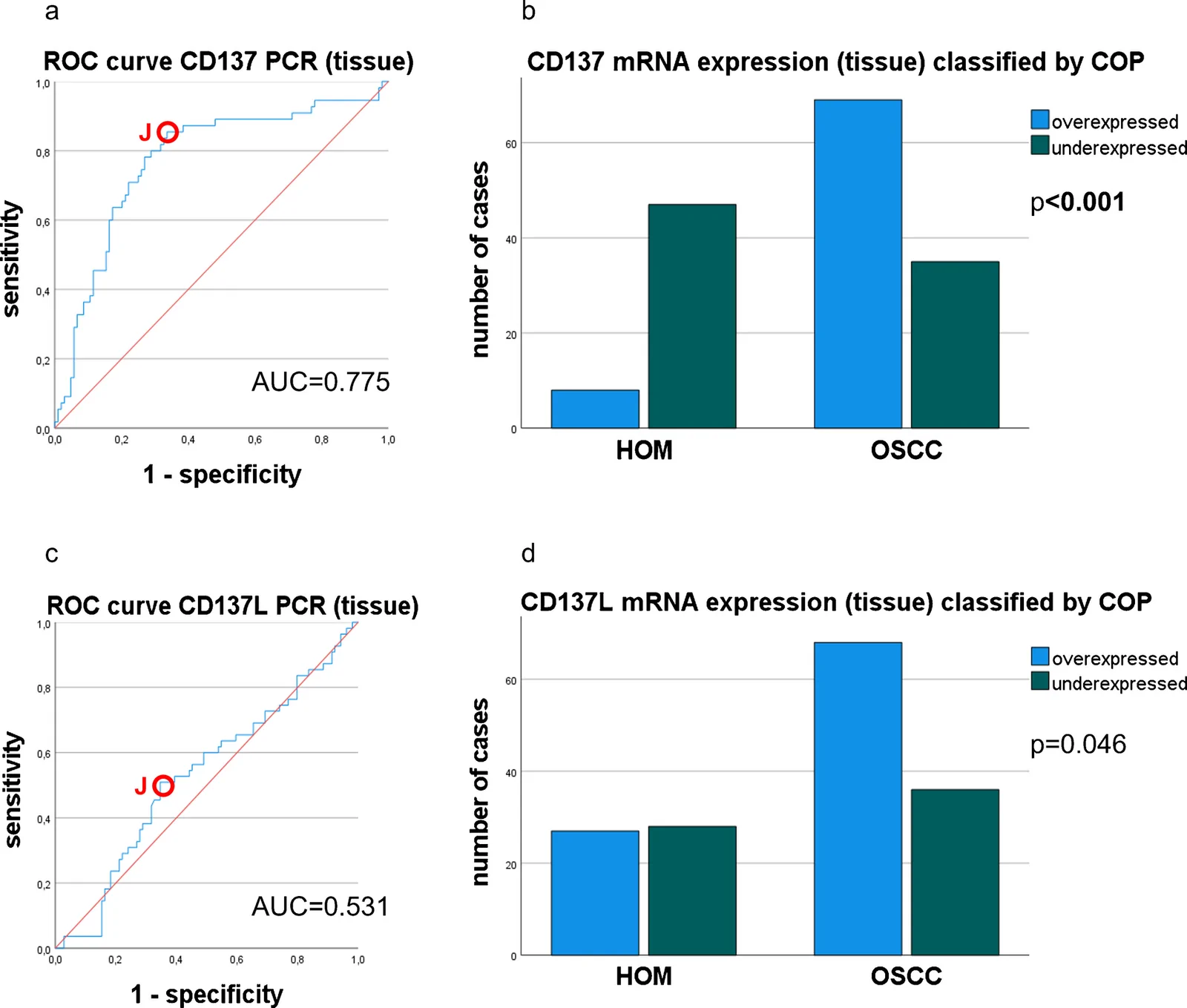
Receiver Operating Characteristic (ROC) curves of CD137 and C137L mRNA expression with determination of cut-off points (COP) (**a**,**c**) and distribution of RT-qPCR tissue samples (HOM vs. OSCC) with reference to the COP (**b**,**d**). The ROC curves illustrate the False Positive Rate (FPR = 1-specificity, X-axis) in relation to the True Positive Rate (TPR = sensitivity, Y-axis) at varying ΔCT thresholds. The given area under the curve (AUC) values provide a quantitative metric for the overall accurancy of the RT-qPCR assay in differentiating between samples from the OSCC and control groups. The optimal threshold that allows for distinction between both groups (HOM vs. OSCC) is indicated by the Youden index (J, Fig. 4a,c). The right-hand side of the figure displays the patient distributions according to the the calculated COP. The corresponding p-values were calculated using the Chi-square test (χ^2^-test). Statistically significant results are indicated by bold font

To substantiate the significance of CD137 upregulation, a ROC curve was formulated from both the RT-qPCR and the IHC data, enabling calculation of the area under the curve (AUC) as previously delineated. For the ROC curves derived from the RT-qPCR analysis, AUC values were 0.775 for CD137 and 0.531 for CD137L. Based upon the data retrieved from the ROC curves, Youden indices (J) were determined from all ROC curves to detect the optimal COP for each curve using the Chi-Square Test. Within the RT-qPCR subcohort, calculated highest J was 0.518 for the CD137 and 0.163 for the CD137L analysis (Fig. 4). The consequently estimated threshold for optimal ΔCT to distinguish between OSCC and HOM (COP) was estimated at 6.84 for CD137 (*p* < 0.001) and 12.73 for CD137L (*p* = 0.046).

**Fig. 5:**
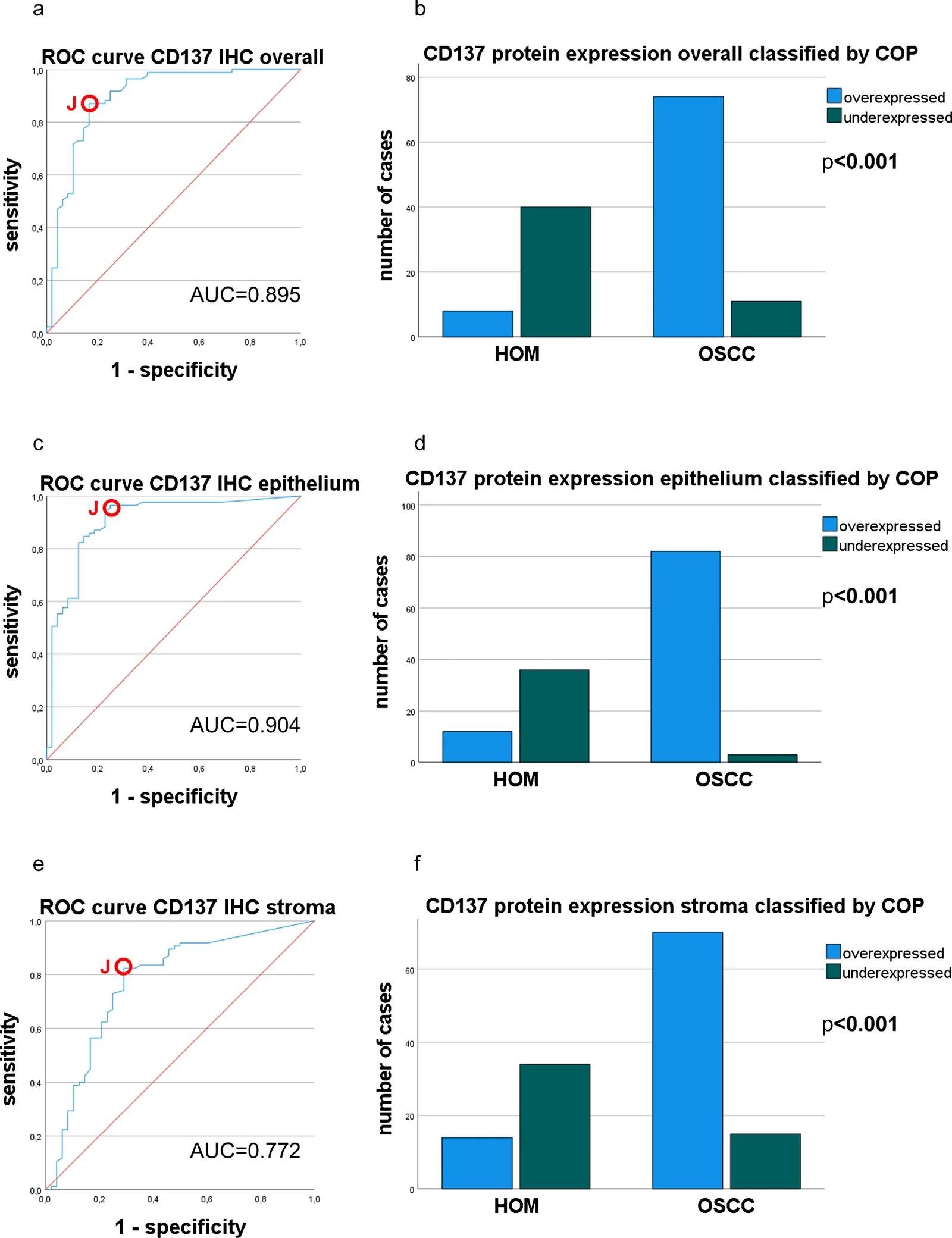
Receiver Operating Characteristic (ROC) curves of CD137 protein expression with determination of cut-off points (COP) (**a**,**c**,**e**) and distribution of IHC tissue samples (HOM vs. OSCC) with respect to the COP (**b**,**d**,**f**). The ROC curves illustrate the False Positive Rate (FPR = 1-specificity, X-axis) in relation to the True Positive Rate (TPR = sensitivity, Y-axis) at different labeling index (LI) thresholds. The reported area under the curve (AUC) values provide a quantitative metric for the overall accurancy of the IHC assay in differentiating between OSCC and control samples. The optimal threshold to discriminate between both groups (HOM vs. OSCC) is indicated by the Youden index (J, Fig. 5a,c,e). The right-hand side of the figure displays the patient distributions according to the the calculated COP. The corresponding p-values were calculated using the Chi-square test (χ^2^-test). Statistically significant results are shown in bold

Within the IHC cohort, calculated AUC values were 0.904 for the epithelial compartment, 0.772 for the stromal compartment and 0.895 for OSCC tissue overall. Consequently, the highest calculated value of J was 0.715 for the epithelial compartment, 0.532 for the stromal compartment and 0.704 for OSCC tissue overall. The corresponding optimal COP regarding the LI was established at 0.185 (*p* < 0.001) in the epithelial compartment, 0.455 (*p* < 0.001) in the stromal compartment and 0.425 (*p* < 0.001) in OSCC tissue overall (Fig. 5).

### Comparison of CD137 and CD137L expression in peripheral blood samples of OSCC and HOM patients

RT-qPCR analysis performed on blood samples from OSCC and HOM patients revealed no statistically significant differential expression of either CD137 or CD137L at mRNA level (Fig. 3c, d). Precisely, mean ΔCT for CD137 was of 5.2 in the OSCC versus 5.21 in the HOM subgroup (FC = 1.01, *p* = 0.653). For CD137L, mean ΔCT was of 13.65 in the OSCC versus 13.7 in the HOM subgroup (FC = 1.035, *p* = 0.351). Given the absence of significant upregulation for both CD137 and CD137L mRNA in the examined blood samples, further ROC analysis was not performed on these groups.

### Association of CD137/CD137L expression in tumor tissue and peripheral blood with histopathological and demographic characteristics

The determined values from both RT-qPCR and IHC were further analyzed referring to the statistical correlation with histopathological parameters such as T-, N-, L-, Pn-status, grading and recurrence status. The analyses were performed using the Mann–Whitney U test and the Kruskal–Wallis test, where applicable. A statistically significant correlation was detected between CD137 tissue expression in RT-qPCR and Pn-status (*p* = 0.044). Furthermore, CD137 mRNA blood expression rates correlated with N-status (*p* = 0.032). For all remaining parameters, no statistically significant correlations were detectable (Table 2).

**Table 2:**
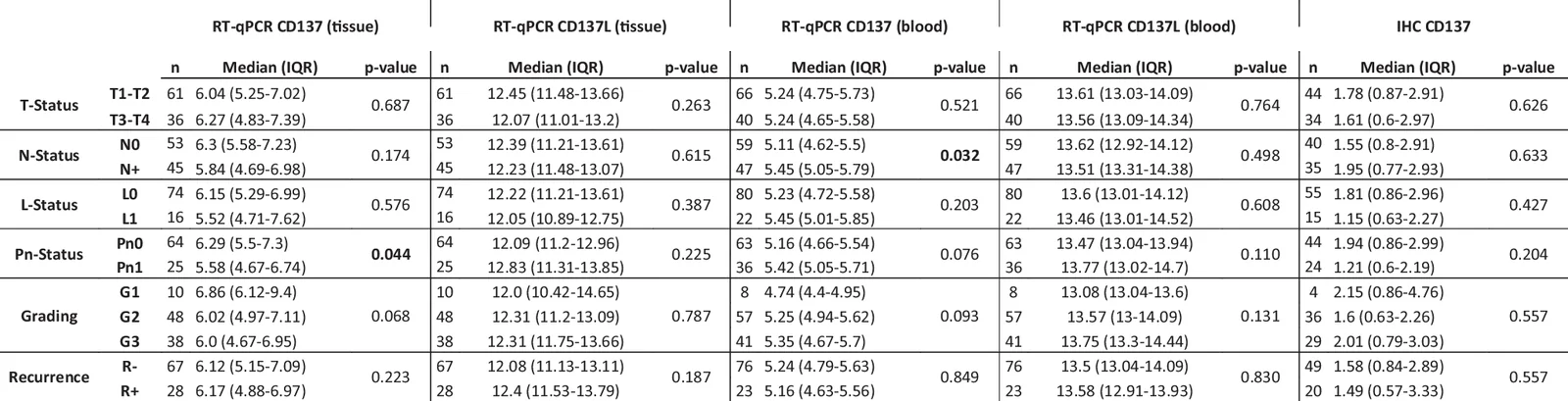
CD137 and CD137L expression in OSCC tissue and blood, quantified via RT-qPCR and IHC, in relation to histopathological characteristics (T-, N-, L-, Pn-statusand grading) and reccurence rate within the study cohort. Median ΔCT values are specified for the RT-qPCR group and median labeling index for the IHC cohort, together with the corresponding interquartile range (IQR). The p-value was assessed via Mann-Whitney U test or Kruskal-Wallis test, where applicable. Statistically significant results are depicted in bold numbers

To evaluate the influence of the age discrepancy between the OSCC and HOM subgroups as a potential confounder on the observed overexpression of CD137 at the mRNA and protein levels, a multivariable linear regression model was employed. This model incorporated patient’s age, diagnostic group and CD137 expression as independent variables. Within the RT-qPCR cohort, OSCC diagnosis remained independently associated with increased CD137 expression (B = −1.57, p < 0.001), while age demonstrated no significant effect (*p* = 0.401). Due to a right-skewed distribution of the protein expression data, the CD137 labeling index was log-transformed prior to multivariable linear regression analysis. In this test, OSCC diagnosis demonstrated a persistent significant association with elevated CD137 expression (B = 0.717, p < 0.001) after statistical adjustment for age (*p* = 0.802).

## Discussion

### Clinical relevance of the study

As IT employing PD-1 inhibitors has rapidly gained in significance in the recent guidelines for OSCC treatment, response rates still remain poor [6, 7, 25]. Further factors for enhancement of IT effectivity and optimal patient selection are consequently desired [26, 27]. Apart from optimal patient selection and timing of IT initiation, a third and probably most promising factor must be taken into consideration when aiming to elevate treatment response rates of IT against OSCC. The factor in question refers to the tumor’s molecular expression patterns and the identification of novel biomarkers, which facilitate introduction of additional immunotherapeutic agents and allow their combination.

The paramount intention of the presented study was therefore to analyze, whether the immune checkpoint CD137 and its ligand CD137L are differentially expressed in OSCC tissue and if the expression patterns of these markers show an impact on clinical presentation of disease.

### Summary of main findings and interpretation in context of existing literature

The study showed significantly increased expression of CD137 in OSCC tissue compared to HOM. The differential expression was highly significant both on mRNA (RT-qPCR) and protein level (IHC). CD137 expression on protein and mRNA level significantly correlated with each other. When subdivided into epithelial and stromal compartment, CD137 expression was only significantly upregulated in the epithelium. Conversely, no statistically significant differential mRNA expression was observed for either CD137 or CD137L in the peripheral blood of the examined patients. With exception of the statistically positive correlation between CD137 mRNA tissue expression and Pn status as well as CD137 mRNA expression in blood and N Status, no further histopathological parameters significantly correlated with either CD137 or CD137L expression.

A recent study by Bin-Alee et al. confirmed high CD137 expression rates in tumor infiltrating lymphocytes (TILS) within various entities of HNSCC [28]. In this examination, particularly high CD137 expression was demonstrated in oropharyngeal and oral cancer, which is in line with the findings of the present study. These observations, at first glance, seem to be contradictory to the widely spread concept of cancer immune evasion. According the immune escape theory, as firstly introduced by Schreiber et al., infiltration of immune cells and expression of co-stimulatory molecules such as CD137 is expected to be rather low in OSCC tissue to enhance tumor cell proliferation [29]. However, one important factor should be carefully considered: CD137 is a protein expressed on a variety of immune cells beyond cytotoxic T cells, with corresponding effects due to cell type and location. A further important immune cell type expressing CD137 for instance are regulatory T cells (T_reg_ cells), a specialized subset of CD4^+^ T helper cells [30, 31]. T_reg_ cells play a pivotal role in the adjustment of immune response, mainly by restriction of overshooting immune reactions and stabilization of immunological self-tolerance [32]. Increased infiltration of T_reg_ cells was observed in a multitude of solid cancer entities and was associated with poor outcome [33–35]. High abundance of T_reg_ cells was also confirmed in peripheral blood and tumor tissue of HNSCC patients, however with heterogeneous results regarding their impact on patient prognosis [36–39]. One possible explanation for these inconclusive results is hypothesized by the still missing clear molecular signature to identify suppressive T_reg_ cells [40]. CD137 recently gained attention as a signature marker for T_reg_ cells with particularly immunosuppressive properties [41]. Nowak et al. identified a strong positive correlation between CD137^+^ expression and T_reg_ cell phenotype in peripheral blood mononuclear cells gained from healthy test patients [42]. Additionally, Forkhead-Box-Protein P3, a transcription factor specifically associated with T_reg_ cells, was proven to upregulate CD137 expression upon T_reg_ activation [43]. A further study performed by Luu et al. discovered, that CD137^+^ T_reg_ cells are able to directly bind to the CD137L expressed on NK cells, thereby reducing their capacity to activate effector T cells through internalization of the CD137/CD137L complex [44]. Moreover, intratumoral T_reg_ cells were proven to upregulate several additional suppressive immune checkpoints associated with immune escape such as TIGIT, OX40, CTLA-4, PD-L1 and PD-L2 [45]. This is one possible explanation, why CD137^+^ expression is associated with better treatment response to Pembrolizumab treatment, as confirmed by Cirillo et al. in a cohort of 40 patients with recurrent or metastatic HNSCC [46].

In this study, increased CD137 expression has not only been confirmed in immune cells within the TME that show T cell like morphology but also by OSCC cells themselves. This observation is in line with the findings of Grimmig et al., who proved elevated expression levels of CD137 and CD137L in multiple colon cancer cell lines [47]. Moreover, high CD137 expression was confirmed to be significantly associated with disease progression and occurrence of metastases, thereby representing a particularly aggressive cancer cell subgroup [47]. In a further study published by Palma et al., expression of CD137 in human leukemia cells was associated with higher proliferative capacity and survival of the malignant cells [48]. The specific mechanisms underlying this elevated level of aggressiveness of cancer cells expressing CD137 however are still insufficiently understood. One possible explanation for this phenomenon might be that expression of an ectopic CD137 complex on cancer cells leads to competitive binding to CD137L on APCs, thereby impairing their capacity of T cell activation [49]. The same, CD137L-neutralizing effect can be achieved through secretion of soluble CD137 by cancer cells [49]. Both phenomena have been described as immune evasion mechanisms in Hodgkin’s lymphoma [50]. Similar tumor promoting effects have been observed in solid cancers such as hepatocellular carcinoma [51]. These findings underline the apparently bidirectional signaling of the CD137/CD137L pathway. When expressed by tumor cells, CD137 also induces regulatory mechanisms, thereby ultimately supporting immune evading effects.

### Strengths and limitations of the study

The current study analyzed a large, heterogeneous number of paired tissue and blood samples from patients suffering from OSCC compared to healthy controls. Tissue samples were analyzed at the mRNA and protein levels using two different methods with equivalent results, underscoring the reliability of the data. By analyzing complementary blood samples, this study allows evaluation of local and systemic expression levels of CD137/CD137L in OSCC. Since the histopathologic assessment and the evaluation of the IHC and RT-qPCR results were performed and double-checked by the same group of scientists, inter-investigator caused bias in the interpretation of the data was minimized.

Limitations include the partially subjective interpretation of the IHC data. Although total cell count of each ROI was performed using a fully-automated procedure, quantification had to be performed manually, thereby allowing a potential observer-related bias. Moreover, neither RT-qPCR nor the single-staining IHC protocol allowed to further specify between the cell types expressing CD137 and/or CD137L. Therefore, it has to be stated that the conclusions derived from the present study are purely descriptive and lack possibilities of a further mechanistic insight into the carcinogenesis and progression of OSCC.

### Conclusions and prospects

In this study, significant overexpression of CD137 in OSCC tissue was detected compared to healthy controls. In contrast, CD137L showed no significant difference in expression. These findings were observed both on mRNA and protein levels, thus emphasizing the relevance of CD137 as a marker worth verifying for immunotherapy applications in OSCC. Although CD137 is generally associated with induction of effector functions, especially activation of cytotoxic T cells, its role appears to be more complex in the TME. When expressed in malignant tumors, CD137 seemingly also mediates regulatory functions and even immunosuppressive effects, depending on the cell type. The distinct effects of CD137 activation are therefore context-dependent and many details of the bidirectional signaling pathway obviously still need to be elucidated. To further quantify the various CD137/CD137L expressing cell types, a subsequent study employing multiplex immunofluorescence staining is currently being prepared by our research group. These findings will facilitate the distinction of the overexpressing cells within the TME, thereby enabling a more precise evaluation of the checkpoint’s biological relevance in OSCC progression and as a target for immunotherapies.

## Supplementary Information

Below is the link to the electronic supplementary material.

ESM 1 (DOCX 12.9 KB)

## Data Availability

Primary data are available upon request from the corresponding author.
