# Supplementary material for: Expression of the immune checkpoint CD137/CD137L in oral squamous cell carcinoma (OSCC) and association with histopathological parameters
Source: Virchows Arch. 2026 Feb 24;489(4):841–53. doi: 10.1007/s00428-026-04430-4 (PMC13714162; doi:10.1007/s00428-026-04430-4)
Supplement: Supplementary file 1 [file 428_2026_4430_MOESM1_ESM.docx]

**Supplementary Table**

| Primer | Sequence (5’ to 3’) | Primer (bp) | Amplicon (bp) | Accession Number |
| --- | --- | --- | --- | --- |
| CD137 p2 s | ACGGGGCAGAAAGAAACTCC | 20 | 99 | NM_001561.4 |
| CD137 p2 as | TCTGGAAATCGGCAGCTACA | 20 |  |  |
| CD137L s | GAGCTTTCGCCCGACGAT | 18 | 114 | NM_003811.3 |
| CD137L as | GTCACTGTACCAGCTCAGGG | 20 |  |  |

**Supplemantary Table 1.** Characteristics of the RT-qPCR primers analyzed in the study.

Primers are listed with the referring base sequence in 5' to 3' order, number of base pairs

(bp), lenght of the produced amplicon and accession number. Annealing temperature for

all primers was 60 °C and 40 cycles were performed for each primer.
